# Mechanism of baixiangdan capsules on anti-neuroinflammation: combining dry and wet experiments

**DOI:** 10.18632/aging.204934

**Published:** 2023-08-08

**Authors:** Qingying Yu, Molin Liu, Tingting Zhao, Mengyue Su, Shukun Wang, Wenhua Xu, Shuhua He, Kejie Li, Xiangyu Mu, Jibiao Wu, Peng Sun, Feng Zheng, Ning Weng

**Affiliations:** 1 College of Pharmacy, Shandong University of Traditional Chinese Medicine, Jinan 250000, China; 2 College of Foreign Languages, Shandong University of Traditional Chinese Medicine, Jinan 250000, China; 3 Preventive Treatment Center, Shenzhen Integrated Traditional Chinese and Western Medicine Hospital, Shenzhen 518000, China; 4 Department of Psychiatry, Boai Hospitai of Zhongshan, Zhongshan 528400, China; 5 Experimental Center, Shandong University of Traditional Chinese Medicine, Jinan 250000, China; 6 College of Traditional Chinese Medicine, Shandong University of Traditional Chinese Medicine, Jinan 250000, China; 7 Innovation Research Institute of Chinese Medicine, Shandong University of Traditional Chinese Medicine, Jinan 250000, China; 8 Department of Neurosurgery, The Second Affiliated Hospital of Fujian Medical University, Quanzhou 362000, China; 9 Department of Traditional Chinese Medicine, Shandong Mental Health Center, Shandong University, Jinan 250000, China

**Keywords:** neuroinflammation, baixiangdan capsule, network pharmacology

## Abstract

Neuroinflammation plays an important role in the pathogenesis of neurological disorders, and despite intensive research, treatment of neuroinflammation remains limited. BaiXiangDan capsule (BXD) is widely used in clinical practice. However, systematic studies on the direct role and mechanisms of BXD in neuroinflammation are still lacking. We systematically evaluated the potential pharmacological mechanisms of BXD on neuroinflammation using network pharmacological analysis combined with experimental validation. Multiple databases are used to mine potential targets for bioactive ingredients, drug targets and neuroinflammation. GO and KEGG pathway analysis was also performed. Interactions between active ingredients and pivotal targets were confirmed by molecular docking. An experimental model of neuroinflammation was used to evaluate possible therapeutic mechanisms for BXD. Network pharmacological analysis revealed that Chrysoeriol, Kaempferol and Luteolin in BXD exerted their anti-neuroinflammatory effects mainly by acting on targets such as NCOA2, PIK3CA and PTGS2. Molecular docking results showed that their average affinity was less than −5 kcal/mol, with an average affinity of −8.286 kcal/mol. Pathways in cancer was found to be a potentially important pathway, with involvement of PI3K/AKT signaling pathways. In addition, *in vivo* experiments showed that BXD treatment ameliorated neural damage and reduced neuronal cell death. Western blotting, RT-qPCR and ELISA analysis showed that BXD inhibited not only the expression of IL-1β, TNF-α and NO, but also NF-κB, MMP9 and PI3K/AKT signaling pathways. This study applied network pharmacology and *in vivo* experiments to explore the possible mechanisms of BXD against neuroinflammation, providing insight into the treatment of neuroinflammation.

## INTRODUCTION

Neuroinflammation is an inflammatory response within the brain or spinal cord, a complex immune response to various injurious stimuli to the nervous system. This inflammation is mainly mediated by cytokines, chemokines, second messengers and reactive oxygen species (ROS) produced by resident glial cells (microglia and astrocytes) upon activation [1, 2]. Normally, neuroinflammation is a necessary protective response to injurious stimuli to the central nervous system, which helps to clear pathogens and cellular debris, resist injurious stimuli and promote tissue repair and regeneration [2, 3]. However, the action of neuroinflammation is dual in nature, which depends mainly on the intensity and duration of the inflammation. If for some reason the inflammatory response goes excessively uncontrolled or persists over time, it causes pathological neuroinflammation, which increases blood-cerebrospinal fluid barrier permeability and peripheral immune cell infiltration, and elevated levels of classical inflammatory cytokines, leading to cell death and histopathological damage [1, 4]. Neuroinflammation plays a central role in many complex health problems, such as neurodegenerative diseases [5, 6], autoimmune diseases (e.g., multiple sclerosis [7]), infections [8], strokes [9], traumatic injuries [10], inflammatory pain [11], postoperative cognitive impairment [12], epilepsy [13], and depression [14].

Currently, treatment of neuroinflammation includes acupuncture interventions and medication. Although acupuncture therapy is gaining recognition as a natural treatment with a long history of simplicity, effectiveness, and no side effects, it is not always feasible because the mechanism of action of acupuncture for neuroinflammation remains unclear.

Therefore, a variety of related drugs such as NSAIDs and steroidal anti-inflammatory drugs are already available on the market. Minocycline appears to inhibit the pro-inflammatory phenotype of microglia without affecting the beneficial regulatory functions of microglia. Statins have many effects on the central nervous system, including affecting microglia activation, preventing ROS formation, inhibiting matrix metalloproteinases (MMPs) and reducing excitotoxicity, and are able to reduce pro-inflammatory immune responses while enhancing regulatory immune responses [15]. Despite the clear targeting of western drug therapy, which captures the common characteristics to play a therapeutic role, the pure application of western drugs for neuroinflammatory diseases cannot meet the needs of clinical treatment due to the influence of adverse factors such as drug resistance.

In this context, herbal medicine is attracting more and more attention from researchers as a source of novel compounds. A variety of herbal extracts and their monomeric compounds block microglia activation, and herbs or components that follow the efficacy of “tonifying deficiency” [16], “clearing stasis” [17] and “expelling phlegm” [18, 19] all have anti-neuroinflammatory effects. Bai Xiang Dan Capsules (BXD) are made up of a combination of Baishao, Xiangfu and Mudanpi. Its representative components such as total peony glycosides [20, 21], isoeugenolone and tannin have obvious anti-inflammatory and immunomodulatory effects. Studies have shown that PF reduces the secretion of CNS inflammatory factors and inhibits microglia activation in a mouse model of inflammatory pain, thereby inhibiting the Akt-NF-κB signalling pathway to reduce neuroinflammation [22]. PF also inhibits the activation of NOD-like receptor thermal protein domain associated protein 3 (NLRP3) inflammatory vesicles to reduce neuroinflammation [23]. Huang et al. [24] evaluated and investigated the anti-neuroinflammatory effects of α-cyperone, a major constituent of Xiangfu, using an LPS-induced BV2 cell inflammation model and found that it could inhibit cytokine production and exert anti-neuroinflammatory effects in model cells by activating Akt and inhibiting the NF-κB pathway. ZHAO et al. [25] found that tannin may promote the polarization of M2 microglia and inhibit the formation of NLRP3 inflammatory vesicles and cellular scorching by intraperitoneal injection into spinal cord injured rats.

In order to explore the potential mechanisms of action of BXD, a network analysis approach based on “drug-target-disease” interactions was used. In 2007, HOPKINS [26] first introduced the concept of “network pharmacology” and defined it as an emerging discipline that breaking through the traditional concept of “one gene, one drug, one disease” and building a bridge to study the interrelationship between new drug development and drug mechanism of action research. Molecular docking, which is commonly used to calculate the various possible binding conformations between biomolecules and small molecule ligands, construct a receptor-ligand conformation set and predict the strength and type of binding, scoring it according to the ranking of the conformation and energy match, has been used for decades so far [27, 28] and is widely used in CNS disorders [29], restless legs syndrome [30] and depression [31]. Therefore, the study used techniques and methods such as neuroinflammation models and bioinformatics analysis to explore the anti-neuroinflammatory effects and mechanisms of BXD.

## MATERIALS AND METHODS

### BXD active ingredient screening

The constituent herbs of BXD were uploaded to TCMSP (https://tcmsp-e.com/tcmsp.php) for screening of active ingredients [32]. Chinese names “baishao”, “xiangfu” and “mudanpi” were used as keywords for the search. The screening criteria of OB ≥ 30% [33], DL ≥ 0.18 [34] and HL ≥ 4 h [35] were set as the active ingredients.

### Screening of BXD targets for anti-neuroinflammatory action

Potential targets for BXD were obtained from the TCMSP, SwissTargetPrediction Tools (http://www.swisstargetprediction.ch/) [36] and SEA database (https://sea.bkslab.org/) [37] and were identified through the TTD (https://db.idrblab.net/ttd/) [38], drugbank (https://go.drugbank.com/ut/med-search-210910) [39], DisGeNET (https://www.disgenet.org/) [40], GeneCards (https://www.genecards.org/) and OMIM database (https://omim.org/) [41] to obtain neuroinflammation-related targets. The targets were imported into the UniProt database (https://www.uniprot.org/) and all protein targets obtained by retrieval were corrected to Uniprot ID [42]. Potential targets of BXD for neuroinflammation were then obtained from the bioinformatics platform (http://www.bioinformatics.com.cn/).

### Protein-protein interaction (PPI) network construction for intersecting targets

Upload String (https://string-db.org/) of the intersection target to build a PPI network. The protein interaction relationship network and data were obtained and saved in TSV format. The network was imported into Cytoscape 3.8.2 for topological analysis and core therapeutic targets were selected based on parameters such as degree, betweenness centrality (BC) and closeness centrality (CC). PPI networks were visualized and key genes were filtered out by CytoHubba plugin [43, 44]. Define targets that are greater than or equal to the median of the degree values as core targets. The PPI network diagram was reconstructed based on the screening results [45].

### Enrichment analysis of intersecting targets

The anti-neuroinflammatory targets of BXD described above were imported into the David database (https://david.ncifcrf.gov/) with a threshold *P* < 0.05 [46] for biological function and pathway enrichment analysis, and the results were visualized through the bioinformatics platform. The core targets and key pathways are imported into Cytoscape 3.8.2 software to build a “target-pathway” network.

### Molecular docking of potential active ingredients to core target proteins

The structures of the individual compounds screened above were downloaded from the PubChem database. Also, enter the entry number in the RCSB PDB database (https://www.rcsb.org/, AKT1 ID: 6npz, Resolution: 2.12 Å; CYP19A1 ID: 3eqm, Resolution: 2.9 Å; MAPK1 ID: 3w55, Resolution: 3 Å; NCOA2 ID: 3up3, Resolution: 1.25 Å; NFKB1 ID: 7lfc, Resolution: 2.2 Å; PIK3CA ID: 6i1s, Resolution: 1.52 Å; PIK3R1 ID: 3i5s, Resolution: 3 Å; PTGS2 ID: 5f19, Resolution: 2.04 Å; PTPN1 ID: 7mn9, Resolution: 1.24 Å; RELA ID: 2ce2, Resolution: 1 Å) [47] in order to download the 3D molecular structure of the receptor. In this study, we used CBDock 2 (https://cadd.labshare.cn/cb-dock2/php/index.php) [48–50] to perform the docking.

### Materials

Lipopolysaccharide (LPS) (L2880, Sigma-Aldrich); Nitric Oxide (NO) assay kit (A013-2-1, Nanjing Jiancheng Bioengineering Institute); Mouse IL-1β ELISA kit (JYM0531Mo, Wuhan Genome Biotechnology Co., Ltd.); BCA Protein Assay Kit (PC0020, Solarbio); SDS-PAGE Gel Rapid Preparation Kit (G2037-50T, Servicebio); anti-PI3K rabbit antibody (AF6241, Affinity), anti-Phospho-AKT1/2/3 rabbit antibody (AF0016, Affinity), anti-AKT1 rabbit antibody (AF0836. Affinity), anti-NF-kB p65 rabbit antibody (AF5006, Affinity), anti-MMP9 rabbit antibody (AF5228, Affinity), anti-TNF alpha rabbit antibody (AF7014, Affinity) and anti-β-actin rabbit antibody (GB11001, Servicebio); HRP-conjugated goat anti-rabbit IgG (GB23204, Servicebio); Nissl staining solution (G1036, Servicebio); Trizol (15596026, ambion); Oligo (dT)18 Primer (3806, TAKARA), PrimeScript II RTase (2690A, TAKARA) and Recombinant RNase Inhibitor (2313A, TAKARA).

### Animals

Male C57BL6 mice, body mass 18–22 g, purchased from Jinan Penyue Laboratory Animal Breeding Co., Ltd. All mice were housed in environmentally controlled, SPF-rated animal houses. All care and experimental procedures were carried out according to the requirements of the National Institutes of Health Guide for the Care and approved by the Animal Experiment Ethics Committee of Shandong University of Traditional Chinese Medicine (No. SDUTCM20200312008).

### Construction of neuroinflammation model [51]

LPS, a characteristic endotoxin of the outer membrane of Gram-negative bacteria, and acts as a potent immune system stimulant, activating and releasing inflammatory mediators [48], which promote neuroinflammation and lead to programmed cell death of neurons in the early stages of infection [52, 53]. After 1 week of acclimatization, neuroinflammation was induced by intraperitoneal injection of LPS for 1 week. Similarly, BXD was administered orally to observe the protective effect on neuroinflammation. SPF grade 6–8 week male rats were studied and randomly divided into: (1) control: saline (intraperitoneal injection (ip)) and oral administration of distilled water; (2) LPS: LPS (ip, 10 mg/kg/d) [51] and oral administration of distilled water; (3) LPS+BXD: LPS (ip, 10 mg/kg/d) and oral administration of BXD (20 g/kg). At 24 h after the final dose, the mice were anesthetized with isoflurane and the brain tissue was quickly removed and weighed for mass.

### Nissl staining

The presence and disappearance of Nissl body is an important indicator of whether nerve cells are damaged, and when encephalitis, cerebral ischemia, and axonal reactions occur, Nissl body can lyse or even disappear. Mice were anesthetized with isoflurane 24 h after the onset of neuroinflammation, fixed by infusion of paraformaldehyde and brains were rapidly harvested. Brains were serially coronally sectioned and immerse in Nissl staining solution for staining. And analyzed using Image J.

### Measurement of IL-1β and NO

Brain tissue from the hippocampus was rapidly extracted 24 hours after the onset of neuroinflammation. The cytokines were detected according to the ELISA kit instructions and the secretion levels were calculated.

### Western blotting

Brain tissue was rapidly extracted from the hippocampus 24 h after the onset of neuroinflammation. After extraction of total hippocampal protein from the 3 groups, their protein concentrations were assayed with the BCA kit. Blots were transferred to PVDF membranes (Millipore, USA) after SDS-PAGE electrophoresis. Primary antibodies (1:1000) using anti-PI3K, phospho-AKT1/2/3, AKT1, NF-kB p65, MMP9, TNF alpha and β-actin rabbit antibody overnight at 4°C. The secondary antibody was incubated for 1 hour at room temperature and the protein bands were recorded on the gel imaging instrument (Tanon, China).

### Real-time quantitative reverse transcription polymerase chain reaction (RT-qPCR)

RNA was extracted from hippocampal tissue by adding Trizol (Ambion, USA) and the RNA concentration was determined using an ultra-micro spectrophotometer (ALLSHENG, China). Total RNA was reverse transcribed into cDNA and amplified for quantitative analysis using specific primers on a fluorescent quantitative PCR instrument. See Table 1 for primer sequence information.

**Table 1:** Primer sequence information.

**Genes**	**Sequence**
**IL-1β**	Forward: TAACCTGCTGGTGTGTGA
	Reverse: TTCTTCTTTGGGTATTGC
**TNF-α**	Forward: TCTACTGAACTTCGGGGT
	Reverse: GGTGGTTTGTGAGTGTGA
**GAPDH**	Forward: CCTTCCGTGTTCCTAC
	Reverse: GACAACCTGGTCCTCA

### Statistical analysis

**Figure 1:**
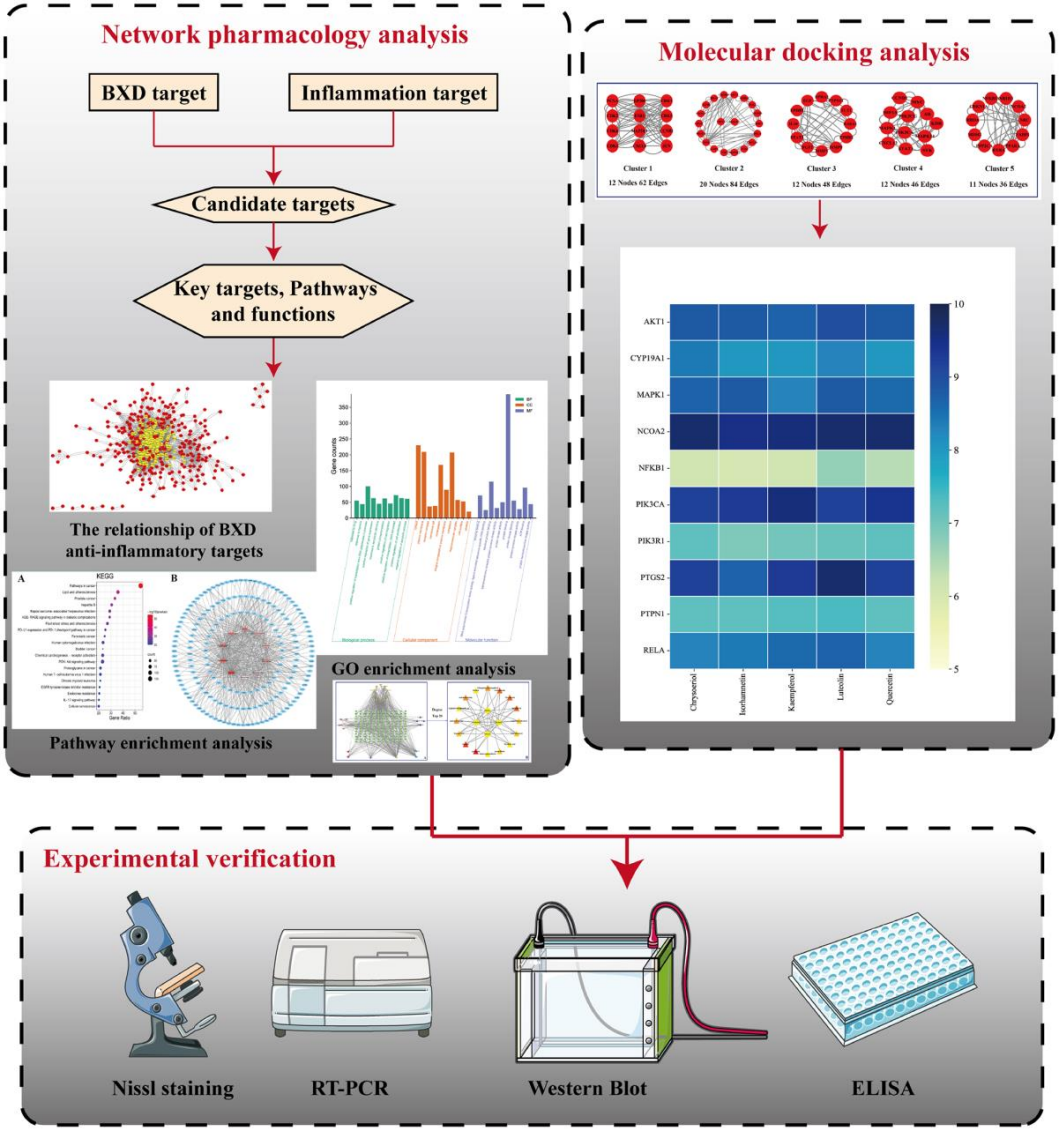
Flow chart of this study.

Measures that conformed to a normal distribution were expressed as mean ± SEM and one-way ANOVA was used for comparisons between multiple groups. Statistical analysis of the data was carried out using Graphpad Prism 8.0 software. A visible graphical summary demonstrating the technical routes is provided (Figure 1).

### Data availability statement

The data used to support the findings of this study are available from the corresponding author upon request. Lead contact, further information and requests for resources and reagents should be directed to and will be fulfilled by Peng Sun, sunpeng@sdutcm.edu.cn.

## RESULTS

### Active ingredients and targets of BXD

The active ingredients of each component of BXD were obtained from the TCMSP database, and a total of 130 active ingredients were obtained after de-duplication (Table 2).

**Table 2:** Active ingredients of BXD.

**Molecular ID**	**Molecule name**	**2D structure**	**OB (%)**	**DL**	**HL**
**MOL001918**	paeoniflorgenone	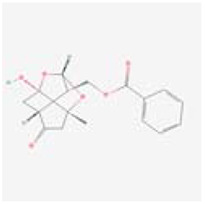	87.59	0.37	7.45
**MOL001919**	(3S,5R,8R,9R,10S,14S)-3,17-dihydroxy-4,4,8,10,14-pentamethyl-2,3,5,6,7,9-hexahydro-1H-cyclopenta[a]phenanthrene-15,16-dione	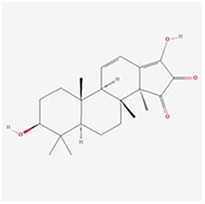	43.56	0.53	4.34
**MOL001921**	Lactiflorin	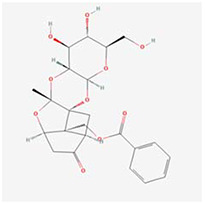	49.12	0.80	7.26
**MOL001924**	paeoniflorin	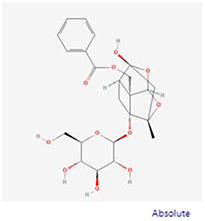	53.87	0.79	13.88
**MOL001928**	albiflorin_qt	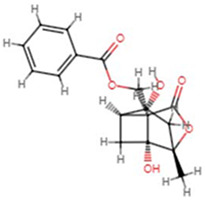	66.64	0.33	6.54
**MOL000359**	sitosterol	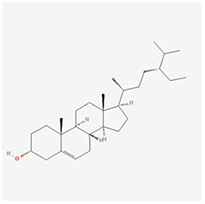	36.91	0.75	5.37
**MOL000422**	kaempferol	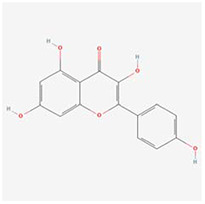	41.88	0.24	14.74
**MOL003044**	Chryseriol	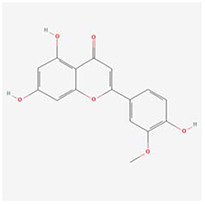	35.85	0.27	16.31
**MOL000354**	isorhamnetin	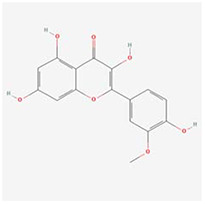	49.6	0.31	14.34
**MOL003542**	8-Isopentenyl-kaempferol	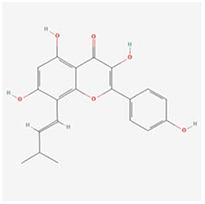	38.04	0.39	15.37
**MOL000358**	beta-sitosterol	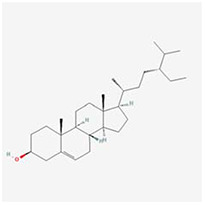	36.91	0.75	5.36
**MOL004058**	Khell	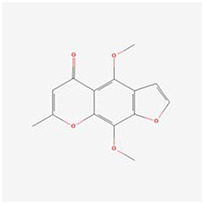	33.19	0.19	10.87
**MOL004059**	khellol glucoside	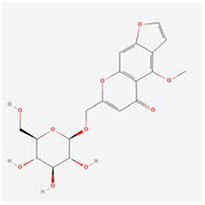	74.96	0.72	14.34
**MOL004068**	rosenonolactone	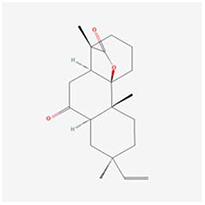	79.84	0.37	6.55
**MOL004074**	stigmasterol glucoside_qt	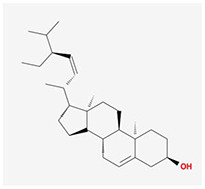	43.83	0.76	5.64
**MOL004077**	sugeonyl acetate	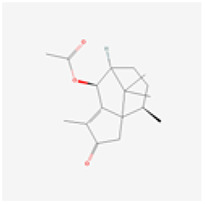	45.08	0.2	4.22
**MOL000449**	Stigmasterol	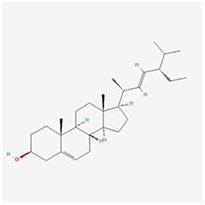	43.83	0.76	5.57
**MOL000006**	luteolin	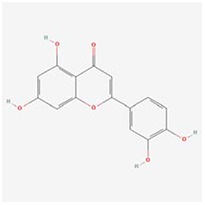	36.16	0.25	15.94
**MOL000098**	quercetin	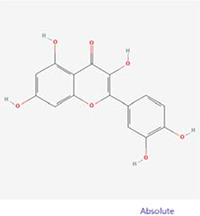	46.43	0.28	14.4
**MOL001925**	paeoniflorin_qt	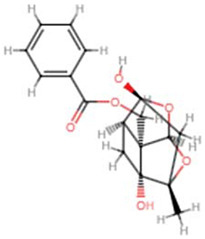	68.18	0.4	8.81
**MOL000211**	Mairin	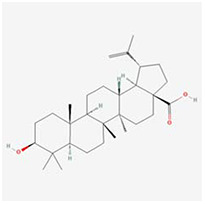	55.38	0.78	8.87
**MOL007003**	benzoyl paeoniflorin	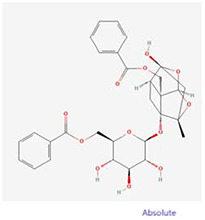	31.14	0.54	15.66
**MOL007369**	4-O-methylpaeoniflorin_qt	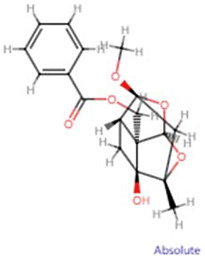	67.24	0.43	9.51
**MOL007382**	mudanpioside-h_qt 2	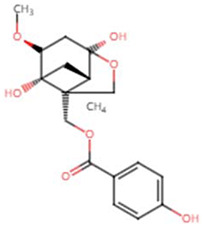	42.36	0.37	7.6
**MOL007384**	paeonidanin_qt	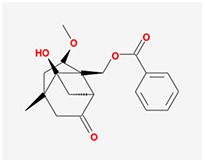	65.31	0.35	7.1

**Figure 2:**
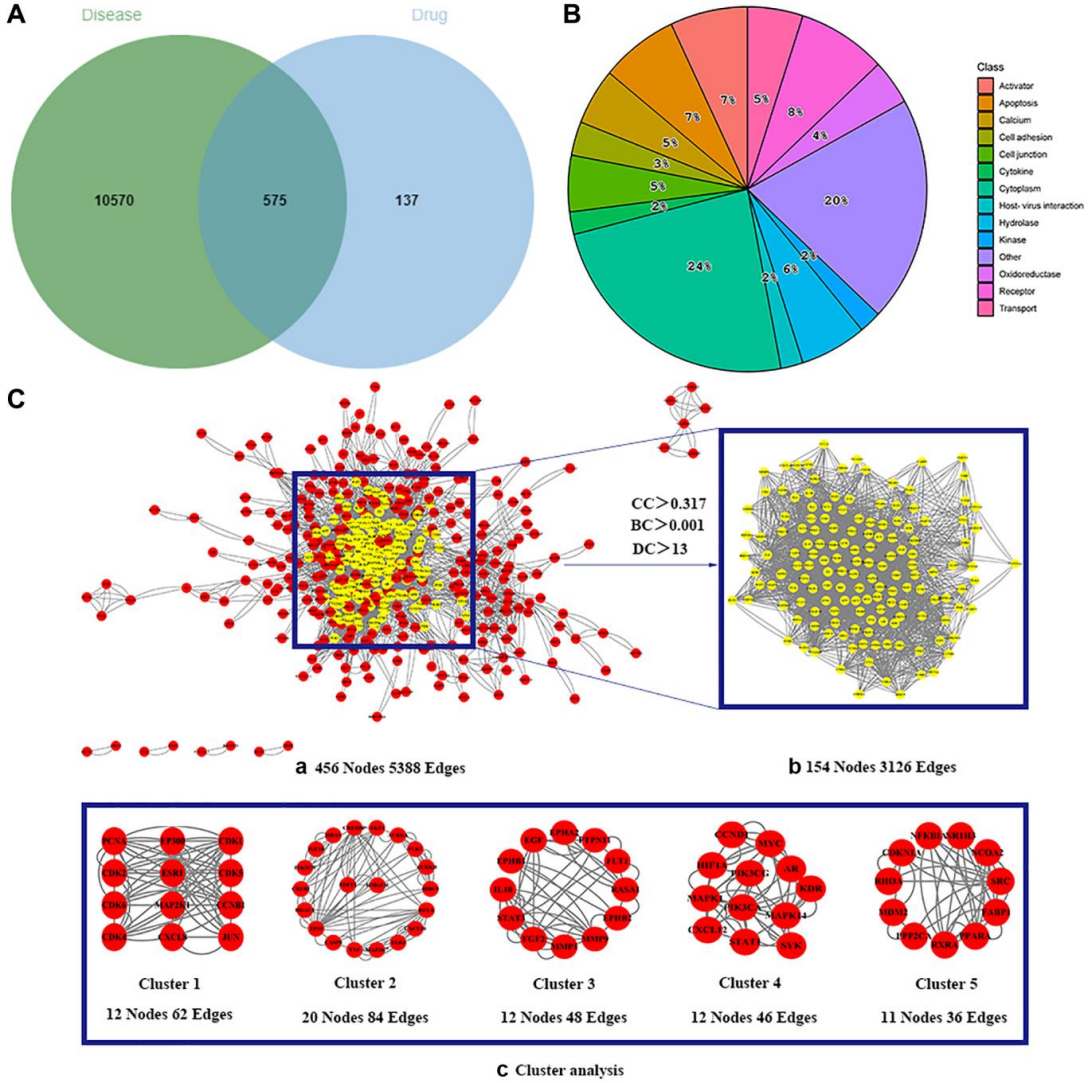
**Identification of potential targets of BXD for the treatment of neuroinflammation.** (**A**) Venn diagram of overlapping targets for BXD and neuroinflammation. (**B**) The BXD anti-inflammatory target classes. (**C**) Identification of candidate targets for BXD against neuroinflammation via PPI analysis. (**a**) PPI networks of shared targets between BXD and neuroinflammation analyzed by STRING 11.5. (**b**) Most significant module identified by the topology selection. (**c**) Core 154 targets in the PPI network based on clustery analysis using the MCODE plug-in.

The 130 active ingredients obtained were searched in TCMSP and 713 drug targets of action were obtained after screening and de-weighting. After removing duplicate targets, a total of 11,145 neuroinflammation-related disease targets were screened using TTD, DisGeNET, Genecards, OMIM and DrugBank disease databases, and the target genes corresponding to BXD were intersected to obtain 575 common gene targets as key targets for the treatment of neuroinflammation in BXD, see Figure 2A. These targets were further divided into fourteen categories (Figure 2B), including oxidoreductase (23 targets), receptor (46 targets), transport (29 targets), activator (40 targets), apoptosos (40 targets), calcium (29 targets), cell adhesion (17 targets), cell junction (29 targets), cytokine (12 targets), cytoplasm (138 targets), host-virus interaction modulator (12 targets), hydrolase (35 targets), kinase (12 targets), other (115 targets). In summary, the active components of BXD can exert anti-inflammatory effects through the modulation of multiple biological pathways in combination. BXD components acted on a total of 575 targets, of which 456 target proteins interacted with at least 1 other target and 119 targets did not interact with any other targets. 5388 interacting edges existed for the 456 target proteins (Figure 2Ca), implying that these target proteins interact with each other more than a random collection of proteins of the same size and distribution, and that these targets may have stronger biological connections. The results of the active ingredient-neuroinflammatory centre target analysis involved a total of 154 nodes and the topological parameter filtering criteria for PPI were set to degree >13, betweenness >0.001, and closeness >0.317 (Figure 2Cb). The target interaction relationships in the PPI network were analysed and detected 5 clusters. Detailed information is shown in Table 3. The targets in cluster 1 are PCNA, EP300, CDK1, CDK2, ESR1, CDK5, CDK6, MAP2K1, CCNB1, CDK4, CXCL8, JUN, which are mainly cell cycle regulators; the targets in cluster 2 are FDFT1, CASP8, AURKA, AKT1, IGF1R, RELA, TLR4, PIK3CB, MAP3K7, TP53, CXCL10, PLK1, CREB1, BIRC5, HRAS, AURKB, TNF, CREBBP, HMGCR, HDAC1, mainly belonging to inflammation-related factors and B-cell receptor signalling pathway processes; targets in cluster 3 are MMP1, EPHB1, FGF2 PTPN11, FLT1, EGF, IL10, RASA1, STAT3, MMP9, EPHA2, EPHB2, mainly belong to cell differentiation and proliferation processes; targets in cluster 4 are KDR, CCND1, MAPK1, CXCL12, SYK, AR, MYC, MAPK14, PIK3CA, HIF1A, STAT1, PIK3CG, mainly involved in the regulation of cellular immunity; targets in cluster 5 are NCOA2, RHOA, RXRA, PPP2CA, FABP1, SRC, NR1H3, CDKN1A, NFKBIA, MDM2, PPARA, mainly belonging to nuclear receptors (Figure 2Cc).

**Table 3:** The clustering information of 161 overlapping targets.

**MCODE**	**Score**	**Nodes**	**Edges**	**Gene symbol**
1	5.636	12	62	PCNA, EP300, CDK1, CDK2, ESR1, CDK5, CDK6, MAP2K1, CCNB1, CDK4, CXCL8, JUN
2	4.421	20	84	AKT1, AURKA, PLK1, AURKB, BIRC5, RELA, CXCL10, TLR4, MAP3K7, TNF, CASP8, TP53, HDACI, CREB1, PIK3CB, IGF1R, HRAS, CREBBP, FDFT1, HMGCR
3	4.364	12	48	EPHA2, PTPN11, FLT1, RASA1, EPHB2, MMP9, MMP1, FGF2, STAT3, IL10, EPHB1, EGF
4	4.182	12	46	CCND1, MYC, HIF1A, PIK3CG, AR, KDR, MAPK1, PIK3CA, MAPK14, CXCL12, STAT1, SYK
5	3.6	11	36	NFKBIA, NR1H3, NCOA2, SRC, FABP1, PPARA, RXRA, PPP2CA, MDM2, RHOA, CDKN1A

### GO function enrichment

**Figure 3:**
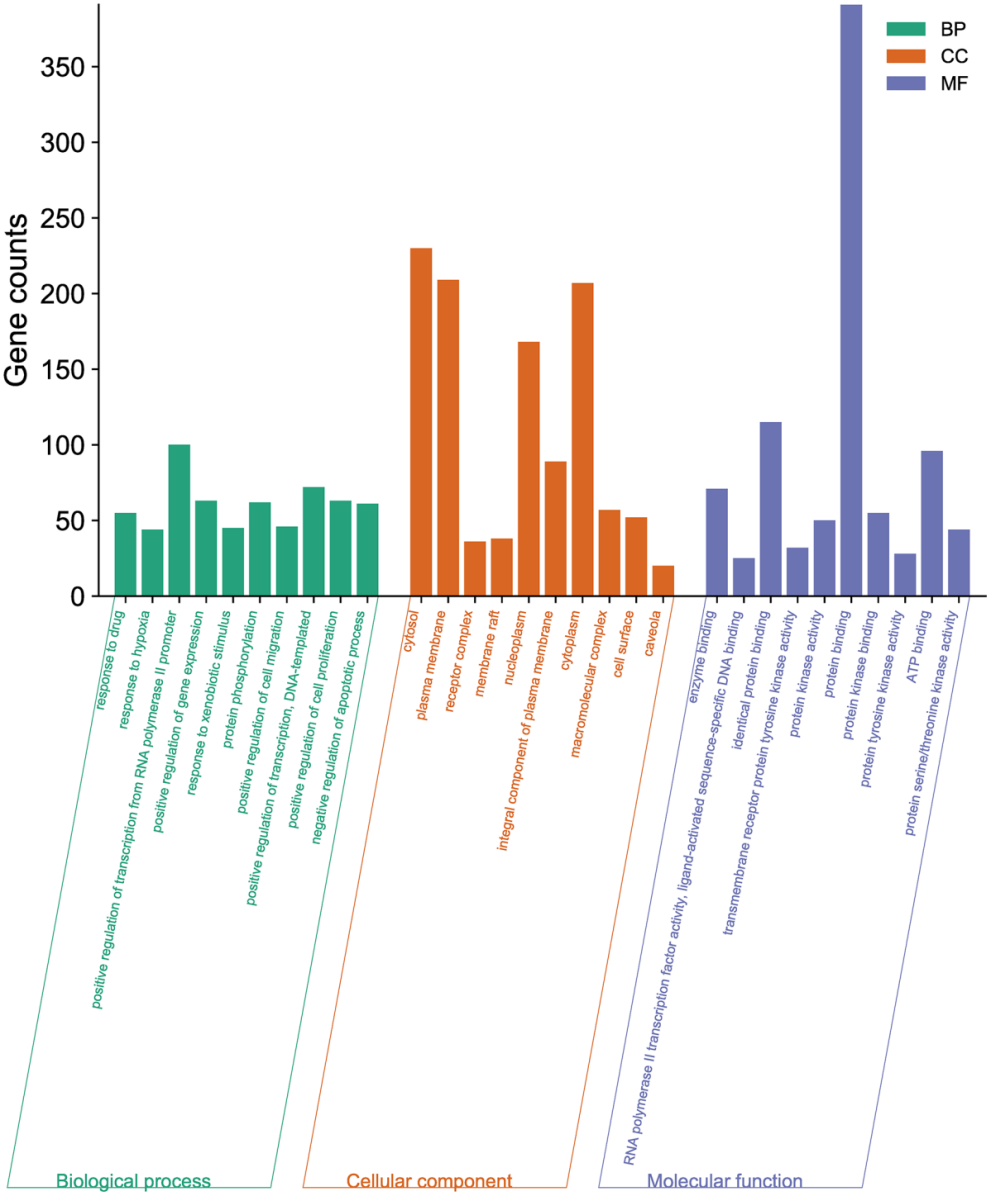
**GO enrichment analysis of the potential targets of BXD against neuroinflammation using DAVID.** Top 10 BP terms, CC terms, and MF terms are shown as green, orange, and purple bars, respectively.

A total of 1807 GO-enriched entries were obtained as a result of the experiment. The top ten BP terms (Figure 3) were response to drug (GO:0042493), response to hypoxia (GO:0001666), positive regulation of transcription from RNA polymerase II promoter (GO:0045944), positive regulation of gene expression (GO:0010628), response to xenobiotic stimulus (GO:0009410), protein phosphorylation (GO:0006468), positive regulation of cell migration (GO:0030335), positive regulation of transcription, DNA-templated (GO:0045893), positive regulation of cell proliferation (GO:0008284), and negative regulation of apoptotic process (GO:0043066). The top ten CC terms (Figure 3) are ranked as follows: cytosol (GO:0005829), plasma membrane (GO:0005886), receptor complex (GO:0043235), membrane raft (GO:0045121), nucleoplasm (GO:0005654), integral component of plasma membrane (GO:0005887), cytoplasm (GO:0005737), macromolecular complex (GO:0032991), cell surface (GO:0009986), and caveola (GO:0005901). The first ten MF terms (Figure 3) are RNA polymerase II transcription factor activity, ligand-activated sequence-specific DNA binding (GO:0004879), protein binding (GO:0005515), identical protein binding (GO:0042802), transmembrane receptor protein tyrosine kinase activity (GO:0004714), protein kinase activity (GO:0004672), protein kinase binding (GO:0019901), protein tyrosine kinase activity (GO:0004713), ATP binding (GO:0005524), protein serine/threonine kinase activity (GO:0004674), and enzyme binding (GO:0019899).

### KEGG pathway enrichment analysis

**Figure 4:**
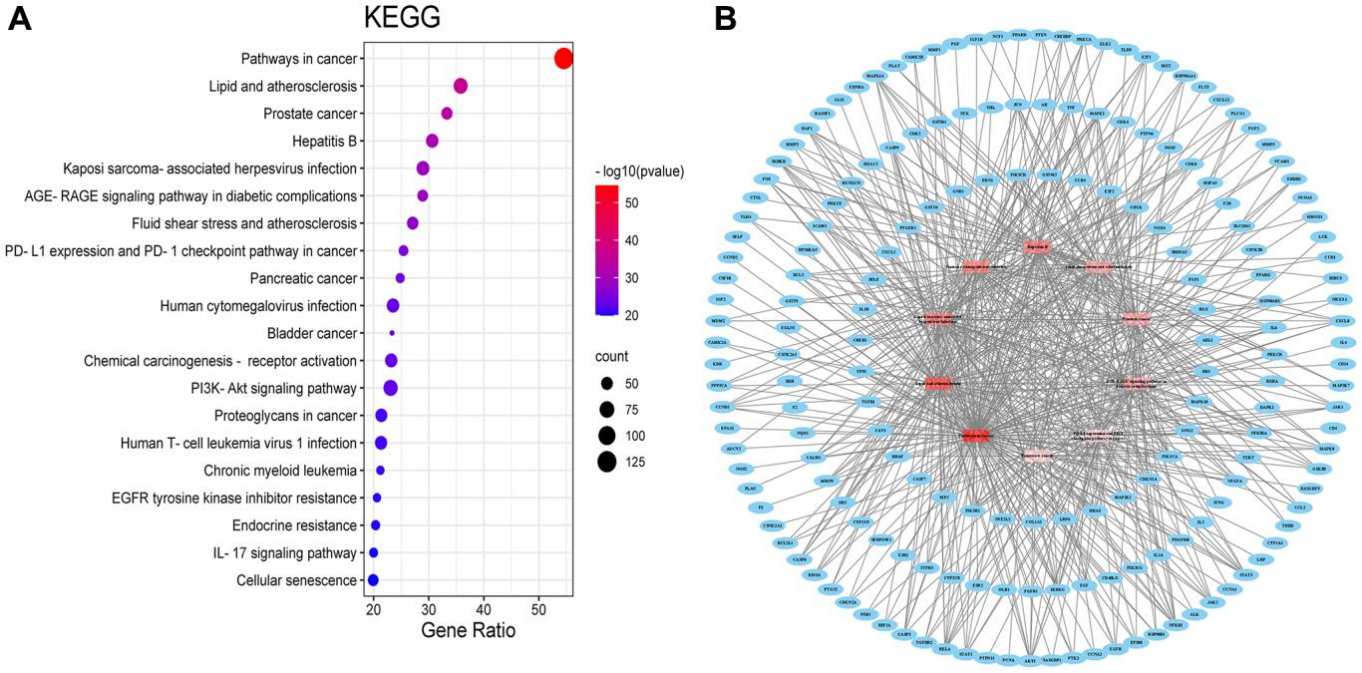
KEGG pathway (**A**) KEGG pathway shown by bubble diagram. (**B**) The Target-pathway network. Blue nodes refer to target proteins. The red nodes represent pathways, and the color red is consistent with the significance of pathways, the deeper red indicates the more significance.

A total of 199 signalling pathways were enriched by KEGG pathway analysis. The BXD anti-neuroinflammation is mainly involved in the following pathways (Figure 4A): Pathways in cancer (hsa05200), Lipid and atherosclerosis (hsa05417), Prostate cancer (hsa05215), Hepatitis B (hsa05161), Kaposi sarcoma-associated herpesvirus infection (hsa05167), interleukin-17 (hsa04657) signaling pathway, AGE-RAGE signaling pathway in diabetic complications (hsa04933), Fluid shear stress and atherosclerosis (hsa05418), PD-L1 expression and PD-1 checkpoint pathway in cancer (hsa05235), Pancreatic cancer (hsa05212), Human cytomegalovirus infection (hsa05163), Bladder cancer (hsa05219), Chemical carcinogenesis - receptor activation (hsa05207), PI3K-Akt signaling pathway (hsa04151), Proteoglycans in cancer (hsa05205), Human T-cell leukemia virus 1 infection (hsa05166), Chronic myeloid leukemia (hsa05220), EGFR tyrosine kinase inhibitor resistance (hsa01521), Endocrine resistance (hsa01522), and Cellular senescence (hsa04218). An analysis of the signalling pathway of BXD in neuroinflammation is shown in Figure 4B.

### Pathways in cancer

**Figure 5:**
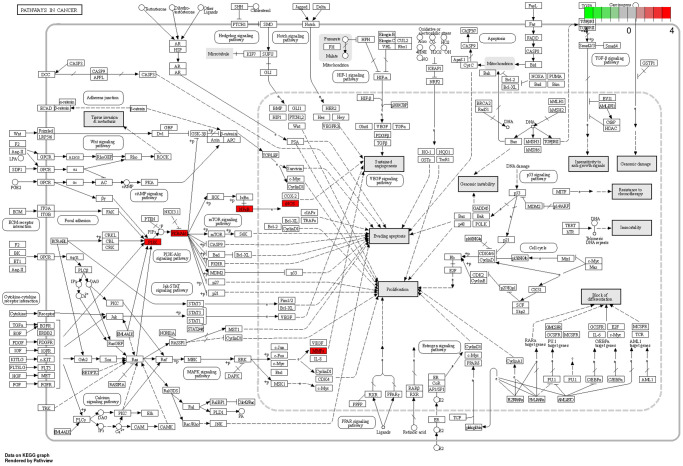
**Pathways in cancer.** The red targets are pivotal genes involved in the therapeutic effects of BXD on neuroinflammation.

Among the ten major signaling pathways, Pathways in cancer (Figure 5) exerted effective anti-neuroinflammatory effects. In Pathways in cancer, BXD may treat neuroinflammation by participating in the PI3K/AKT bioregulatory process.

### Molecular docking experiments

**Figure 6:**
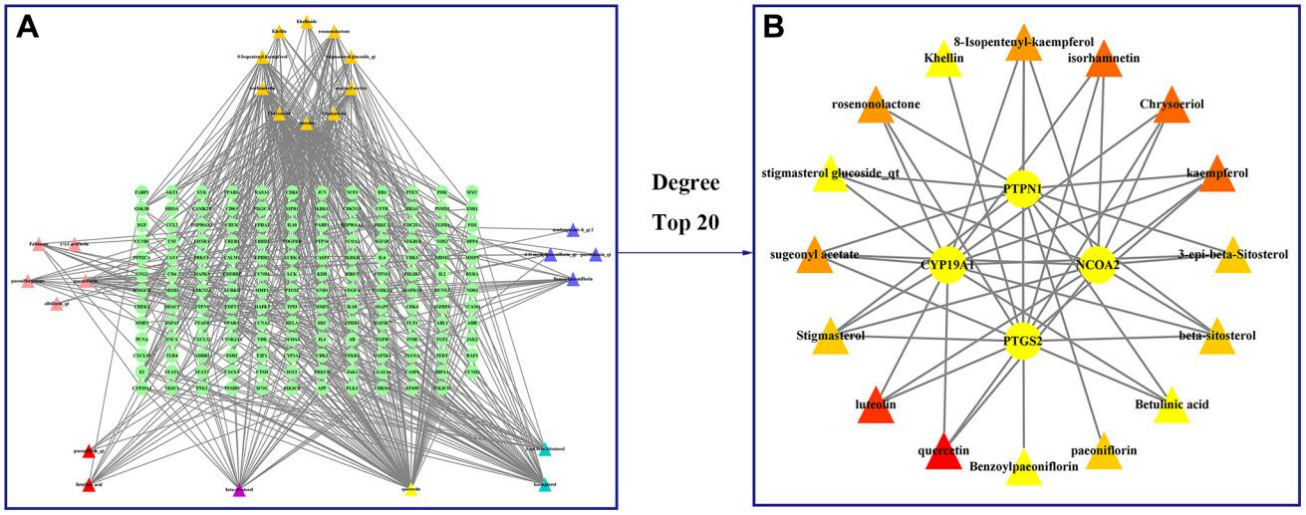
**Construct a drug (BXD component)-disease (neuroinflammation) network.** Nodes representing drug candidates are represented by triangles and targets are represented by circles. (**A**) Drug (component)-disease (neuroinflammatory target) network. (**B**) Select the top 20 nodes from the drug-disease network.

**Figure 7:**
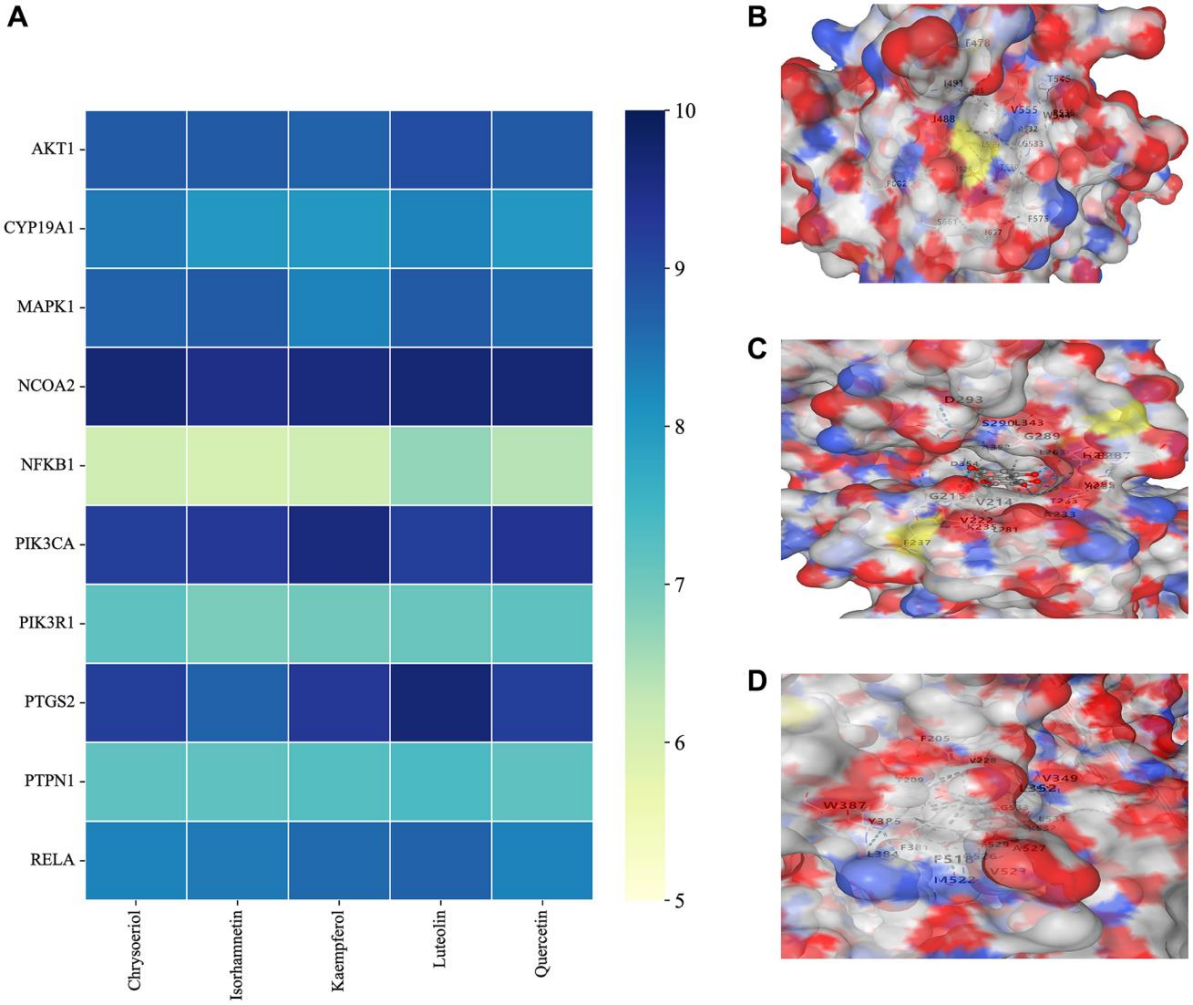
**Molecular docking results.** (**A**) Binding energy values in molecular docking. (**B**) NCOA2 docked with chrysoeriol. (**C**) PIK3CA docked with kaempferol. (**D**) PTGS2 docked with quercetin.

A compound-target network for BXD was constructed using the screened compounds and their targets, see Figure 6A. Each potential target gene interacts with multiple active ingredients, reflecting the multiple pathways, ingredients and targets through which BXD treats neuroinflammation. The higher the degree value, the greater its role in the regulatory network. The top 20 targets and core compounds were mapped using Cytoscape 3.8.2, see Figure 6B. The five core components (chrysoeriol, isorhamnetin, kaempferol, luteolin, and quercetin) obtained during the network graph construction process were first selected. It has been reported that when the molecular docking binding energy is lower, then the small molecule is considered to be able to bind to the protein [54]. Chrysoeriol, kaempferol and luteolin bind strongly to NCOA2, PIK3CA and PTGS2, respectively (Figure 7). They is indeed an effective substance and the main target of action of BXD in the treatment of neuroinflammation.

### Neuroprotective effect of BXD in neuroinflamed mice

**Figure 8:**
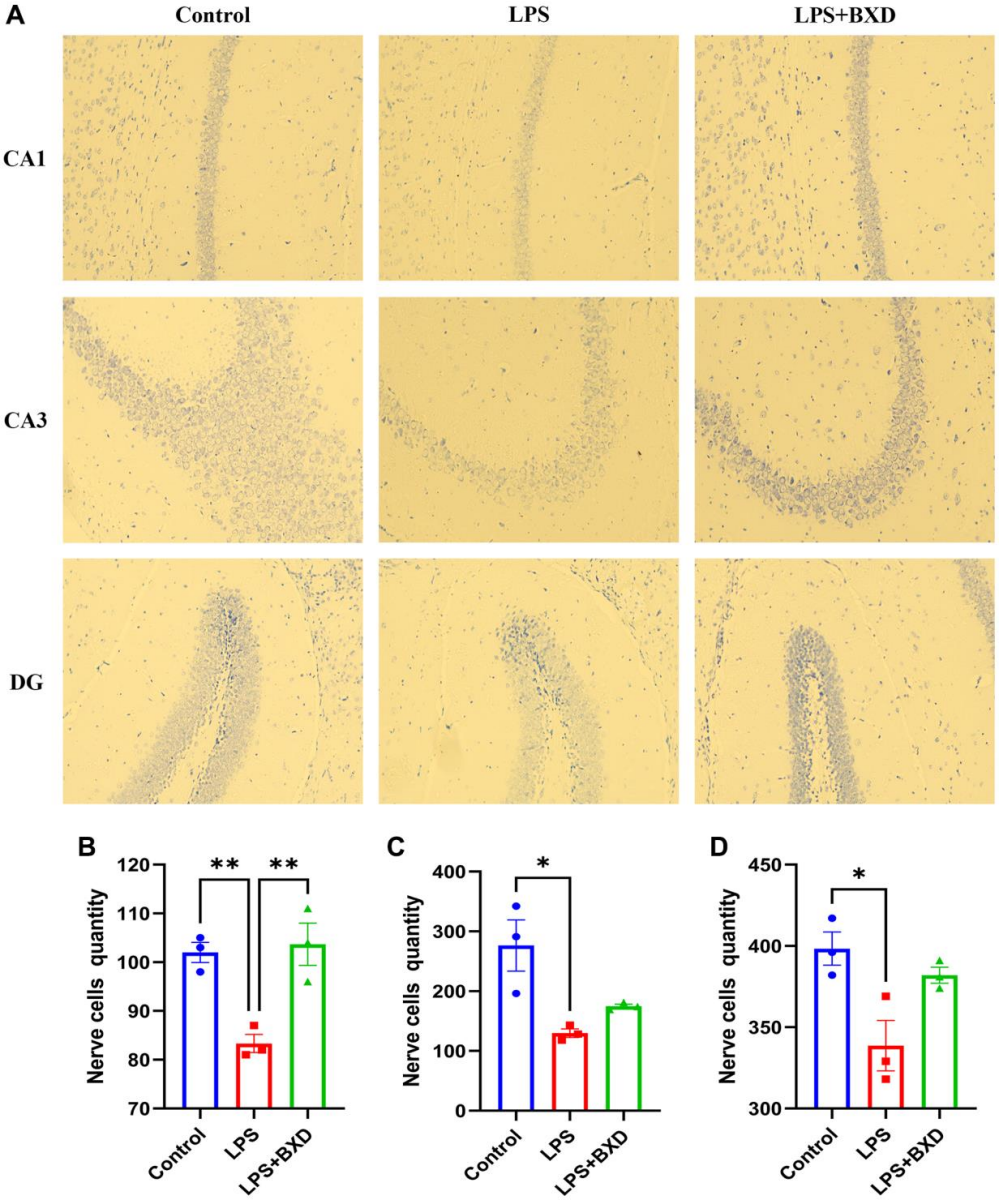
**Nissl staining.** (**A**–**D**) Changes and statistics of Nissl body in the hippocampal CA1, CA3 and DG regions of mice in each group after drug administration. Data represent the mean ± SEM (*n* = 3). ^**^P < 0.01, ^*^P < 0.05, compared with the LPS group.

Microscopically: Figure 8A shows a large number of Nissl body with neat arrangement and regular tight morphology was seen in the Control group. The LPS group showed typical neuropathological changes in CA1 (*P* = 0.0079, Figure 8B), CA3 (*P* = 0.0110, Figure 8C) and DG (*P* = 0.0157, Figure 8D) areas, and the Nissl body positive cell count was significantly reduced compared with the Control group, the difference was statistically significant. the LPS+BXD group significantly improved the histological structure of hippocampal CA1 after gavage of BXD, and the Nissl body positive cell count was significantly increased compared with the LPS group, the difference was statistically significant (*P* = 0.0052).

### Effect of BXD on the PI3K/AKT pathway

**Figure 9:**
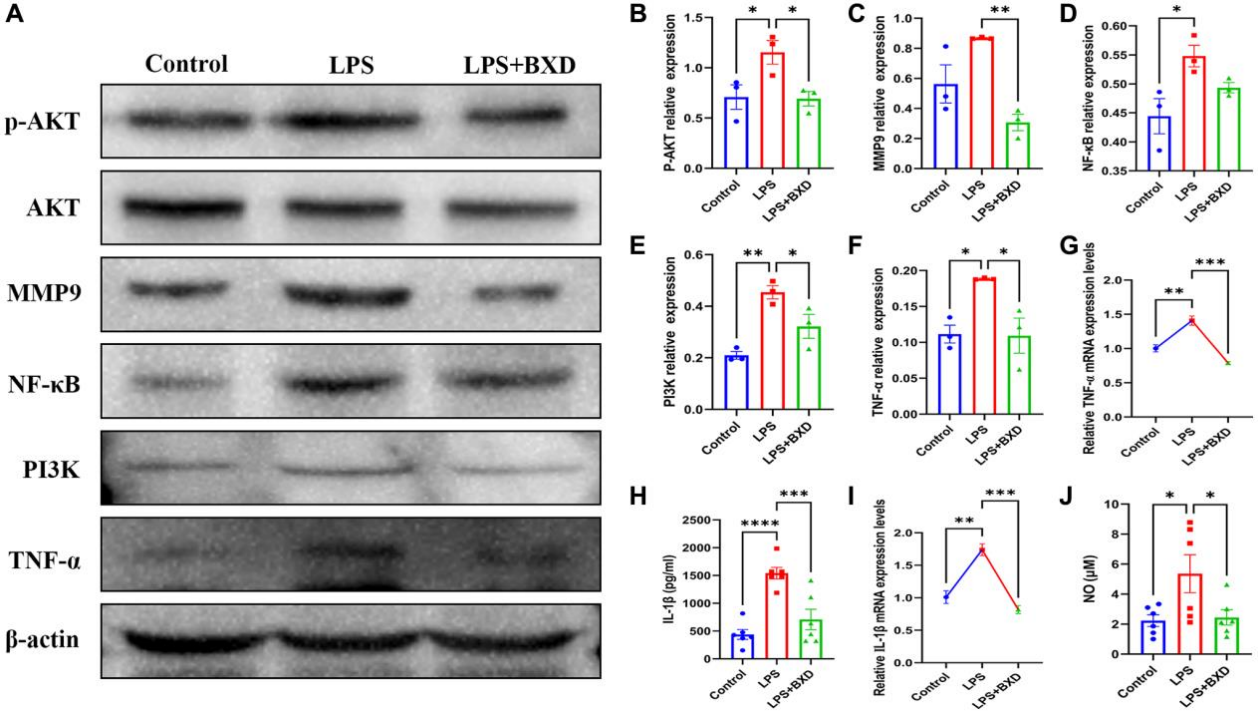
**Effect of BXD on PI3K/AKT pathway and inflammatory factors in LPS-induced neuroinflammation.** (**A**–**F**) Expression levels of PI3K, AKT, P-AKT, MMP9, NF-κB and TNF-α proteins were measured by Western blot, β-actin served as an internal Control. (**G**) The mRNA expression levels of TNF-α was detected by RT-PCR. (**H**) The secretion of IL-1β was detected by ELISA. (**I**) The mRNA expression levels of IL-1β was detected by RT-PCR. (**J**) The secretion of NO was detected by ELISA. Data represent the mean ± SEM (*n* = 3 − 6). ^****^P < 0.0001, ^***^P < 0.001, ^**^P < 0.01, ^*^P < 0.05. Compared with the LPS group.

Figure 9A–9E shows that BXD significantly reduced PI3K/AKT pathway activation induced by LPS. LPS is thought to be a potent trigger for inflammation [55]. Combined WB, PCR and ELISA results revealed that oral administration of BXD significantly ameliorated the increased hippocampal inflammatory factor secretion caused by LPS (Figure 9A, 9F–9J).

## DISCUSSION

Neuroinflammation is a relatively common disease worldwide. Most anti-neuroinflammatory drugs have a single target and are of limited effectiveness. Some traditional Chinese medicines have been shown to have anti-neuroinflammatory effects, however there are fewer studies on the anti-neuroinflammatory effects of BXD. This study uses a network pharmacology approach to initially explore the mechanism of action of BXD in the treatment of neuroinflammation.

The active ingredient-target network shows that BXD is multi-component and multi-target for the treatment of neuroinflammation. The results show that chrysoeriol, isorhamnetin, kaempferol, luteolin, and quercetin are the key active ingredients. Mapping the active ingredients to the common targets of neuroinflammation, 154 common targets of drug-disease action were obtained. Jadaun et al. [56] demonstrated that chrysoeriol can reduce the secretion of pro-inflammatory cytokines through PI3K/Akt/m TOR signaling pathway and inhibit the inflammatory response, thus exerting a neuroprotective function. It has been reported [57] that kaempferol can resist the neuroinflammatory effect induced by 1-methyl-4-phenylpyridinium (MPP+), but its mechanism of action is not clear. Luteolin inhibited the increase TNF-α, IL-6 and NF-κB levels in a high-fat diet mouse model and significantly improved hippocampal and cortical inflammation [58]. Quercetin has significant inhibiting the activation of pro-inflammatory pathways *in vivo* [59].

Pathways in cancer encompass many inflammatory factors that mediate immune responses by a variety of mechanisms, and there are multiple targets in this pathway that are relevant to the pathogenesis of neuroinflammation, such as the Potassium channel AKT1 (AKT1), Nitric oxide synthase, inducible (NOS2), Phosphatidylinositol 4,5-bisphosphate 3-kinase catalytic subunit alpha isoform (PIK3CA), Matrix metalloproteinase-9 (MMP9), Nuclear factor NF-kappa-B p105 subunit (NFKB1), Mitogen-activated protein kinase 1 (MAPK1), and Prostaglandin G/H synthase 2 (PTGS2). This study identified multiple enrichment of all potential target species on pathways in cancer, which may be key pathways for BXD to regulate neuroinflammation.

Activation of AKT in the PI3K/AKT signaling pathway occurs when protein growth factors bind to receptors on the cell surface, inducing phosphorylation of the tyrosine portion of the receptor in the cytoplasm, leading to the aggregation and activation of PI3K [60]. Previous studies have shown that the PI3K/AKT signalling pathway can block the inflammatory response and reduce the release of inflammatory factors to achieve neuronal protection [61], which is generally consistent with the network pharmacology prediction results and *in vivo* anti-inflammatory activity results, indicating that the network pharmacology prediction results have a high degree of accuracy. Zhang et al. [62] demonstrated that the PI3K inhibitor LY294002 abrogated the anti-inflammatory effects of propofol, suggesting that propofol exerts its inhibitory effect on the inflammatory response by modulating this pathway. Lin et al. [63] later found that 8 weeks of swimming training reduced the hyperphosphorylation of PI3K/Akt in the hippocampus of aging rats, attenuated NF-κB activation, reduced the expression of inflammation-related factors and proteins, exerted anti-inflammatory effects, and ameliorated neuronal damage.

Neuroinflammation is an inflammatory response that occurs in the CNS. Excessive production of pro-inflammatory cytokines (NO, iNOS, TNF-α, IL-1β), chemokines (CXCL-8, CCL2, CCL3, CCL4) and ROS cause neuronal and glial cell dysfunction in the form of axonal degeneration, myelin loss and cell death [64]. As an inflammatory factor, LPS induces a stable and easily constructed model of neuroinflammation. In recent years, increasing attention has been paid to the anti-neuroinflammatory effects of herbal medicines. Although existing studies have not used BXD in the treatment of neuroinflammatory diseases for the time being, BXD, as a traditional herbal formulation, is a complementary and alternative medicine to herbal medicines in the treatment of neuroinflammatory diseases. This study found that BXD can affect neuroinflammation through PI3K/AKT signaling pathway, which will surely provide more and more reliable guarantee for the secondary development of BXD and the improvement of clinical efficacy of neuroinflammation.

## CONCLUSIONS

In summary, BXD may exert its anti-inflammatory effects by modulating NF-κB, MMP9, TNF-α, IL-1β, NO and other components of the PI3K/AKT signaling pathway through chrysoeriol, kaempferol and luteolin. This study combines bioinformatics and *in vivo* experiments to identify relevant targets and related pathways of BXD formulations for the treatment of neuroinflammation, and initially reveals the modern value of targeted multi-target therapy of BXD at the molecular level in the study of neuroinflammation.

There are also some shortcomings in this study. It is difficult to fully reflect the overall situation due to limitations such as the lack of comprehensive data in the database and the lack of precision of the action targets obtained. In addition, for complex chemical compounds and multi-targets of BXD, the further study is still needed. In the future, more studies also suggest comparing the changes in PI3K-AKT pathway in the same animals before and after BXD intervention, and the hippocampus of each group of mice could be sampled by microdialysis to further confirm that BXD ameliorates neuroinflammation by altering the PI3K-AKT pathway.
